# Reinitiating antiplatelet therapy in chronic subdural hematoma: Does adjunctive middle meningeal artery embolization improve outcomes?

**DOI:** 10.1007/s10143-026-04318-6

**Published:** 2026-05-09

**Authors:** Muhammed Amir Essibayi, Jay Kakadiya, Hamza Adel Salim, Huanwen Chen, Ahmed Y. Azzam, Adam A. Dmytriw, Vivek S Yedavalli, Mohammad A. Kassar, Marco Colasurdo, Dheeraj Gandhi, Deepak Khatri, Neil Haranhalli, David J. Altschul, Dhairya A. Lakhani

**Affiliations:** 1 Department of Neurological Surgery and Montefiore-Einstein Cerebrovascular Research Lab, Montefiore Medical Center, Albert Einstein College of Medicine, Bronx, NY USA; 2 Department of Neuroradiology, Rockefeller Neuroscience Institute, West Virginia University, Morgantown, WV USA; 3 Department of Neuroradiology, MD Anderson Medical Center, Houston, TX USA; 4 Division of Neurointerventional Surgery, Department of Neurosurgery, University of Maryland Medical Center, Baltimore, MD USA; 5 Nuffield Department of Surgical Sciences, Faculty of Clinical Medicine, University of Oxford, Oxford, UK; 6 Neuroendovascular Program, Women’s Hospital, Massachusetts General Hospital & Brigham, Harvard University, Boston, MA USA; 7 Neurointerventional & Neuroanalytics Collaboration (NAN-C), School of Medicine, Toronto Metropolitan University, Toronto, ON USA; 8 Department of Radiology, Division of Neuroradiology, Johns Hopkins Medical Center, Baltimore, MD USA; 9 Department of Interventional Radiology, Oregon Health and Science University, Portland, OR USA

**Keywords:** Hematoma, Subdural, Chronic, Platelet aggregation inhibitors, Embolization, Therapeutic, Meningeal arteries, Treatment outcome

## Abstract

The optimal timing for reinitiating antiplatelet therapy after treatment of chronic subdural hematoma (cSDH) remains uncertain, especially when middle meningeal artery embolization (MMAE) is used as an adjunct to surgery. This study evaluated the safety and outcomes of early antiplatelet reinitiation in patients undergoing combined surgical evacuation and MMAE, and compared outcomes in antiplatelet-treated patients receiving surgery with versus without MMAE. Adult cSDH patients from the TriNetX database (May 2020–May 2025) were identified using ICD-10 and RXNORM codes. Two propensity score–matched analyses were performed: (1) patients receiving surgery with adjunct MMAE, stratified by antiplatelet initiation within 30 days; and (2) antiplatelet-treated patients undergoing surgery with adjunct MMAE versus surgery alone. Outcomes included rescue surgery and 6-month mortality. After matching, early antiplatelet use in surgery + MMAE patients (n = 163 per group) was not associated with higher rescue surgery rates (OR 0.68, 95% CI 0.32–1.48) or mortality (OR 1.52, 95% CI 0.73–3.20). Among antiplatelet-treated patients, surgery + MMAE (n = 176) had similar rescue surgery rates to surgery alone (n = 176) but significantly lower mortality (10.8% vs. 21.0%; OR 0.46, 95% CI 0.25–0.83, p = 0.009). Early antiplatelet reinitiation appeared safe after cSDH evacuation with adjunct MMAE. The associated lower 6-month mortality with adjunct MMAE in antiplatelet-treated patients is observational and hypothesis-generating, and warrants prospective confirmation.

**Supplementary Information:**

The online version contains supplementary material available at 10.1007/s10143-026-04318-6.

## Introduction

In patients with chronic subdural hematomas (cSDH), antiplatelet medications are frequently discontinued due to concerns regarding hematoma expansion and potential need for additional surgical procedures [1, 2]. This creates a clinical dilemma as these patients face competing thrombotic risks, including ischemic stroke, myocardial infarction, and venous thromboembolism, especially those with underlying cardiovascular disease [3–5]. Currently, there remains significant uncertainty regarding optimal timing for antiplatelet reinitiation in high-risk patients after cSDH treatment [5]. 

Clinical practice surrounding antiplatelet resumption after cSDH evacuation demonstrates considerable heterogeneity, with sparse literature providing definitive recommendations [5, 6]. Substantial variability exists among neurosurgical and neurological practitioners concerning both timing and safety considerations for antiplatelet restart [5]. This clinical uncertainty reflects the paucity of data examining antiplatelet therapy’s influence on hematoma progression, surgical re-intervention rates, and patient mortality [2, 5, 7, 8]. While some investigations have demonstrated comparable outcomes regarding hematoma progression and re-operation rates in patients resuming antiplatelet therapy, the evidence base remains constrained and predominantly derives from traditional surgical cohorts [9]. 

Middle meningeal artery embolization (MMAE) has emerged as an effective intervention that interrupts the cycle of repetitive microhemorrhages, demonstrating superior efficacy in preventing recurrent bleeding and hematoma progression compared to surgical evacuation alone [10–15]. This suggests that patients receiving combined surgical and MMAE treatment may have reduced bleeding risk, potentially creating a safer environment for antiplatelet initiation. Nevertheless, data examining antiplatelet reinitiation in patients treated with combined surgery and MMAE remains extremely limited [9, 16]. A relevant study by Martinez-Gutierrez et al. demonstrated the safety of MMAE in patients requiring ongoing antithrombotic therapy, further supporting the need to investigate antiplatelet timing in this population [17]. 

Given the substantial evidence gap regarding antiplatelet therapy initiation in high-risk patients following combined surgical and MMAE treatment for cSDH, we conducted a comprehensive multi-institutional, multinational retrospective analysis using propensity score matching methodology. Our aim was to evaluate the outcomes of initiating antiplatelet therapy in patients undergoing surgery with adjunct MMAE. Our secondary aim was to compare outcomes between antiplatelet-treated patients receiving surgery with adjunct MMAE versus those receiving surgery alone.

## Methods

This investigation utilized the TriNetX platform (https://trinetx.com/) and adhered to the Strengthening the Reporting of Observational Studies in Epidemiology (STROBE) reporting standards. In accordance with the U.S. Health and Human Services regulations (45 CFR 46) and institutional policy, research using such de-identified datasets does not constitute human subjects research and is therefore exempt from Institutional Review Board or ethics committee approval. As no identifiable patient information was accessed, the requirement for informed patient consent for study participation and for the procedures described was waived. All research activities were conducted in compliance with the principles of the Declaration of Helsinki.

### Study population and inclusion criteria

Patient cohorts were identified using established TriNetX methodologies previously described in the literature [18–22]. Adult patients (≥ 18 years) with cSDH were extracted from the TriNetX database covering the period from May 6, 2020, to May 7, 2025, utilizing International Classification of Diseases, 10th revision (ICD-10) diagnostic codes (**Supplemental Table ****1**). Patients receiving surgical intervention alone or combined with MMAE within 30 days of cSDH diagnosis were identified through ICD-10 Procedure Coding System (PCS) codes. Antiplatelet therapy initiation within 30 days of surgery was identified using RXNORM terminology (Supplemental Table 1).

Two distinct analytical approaches were employed. First, patients receiving combined surgery and MMAE were stratified by whether they were initiated on antiplatelet therapy within 30 days or not. This analysis examined the effects of antiplatelet therapy in patients undergoing combined surgery and MMAE. Second, among patients initiated on antiplatelet therapy, we compared outcomes in those receiving surgery with adjunct MMAE with those receiving surgery alone. This comparison assessed how MMAE impacts outcomes for patients on antiplatelet therapy.

### Data source

The TriNetX research platform (Cambridge, MA) encompasses over 275 million patient records from 120 healthcare institutions spanning 30 countries. The platform provides real-time access to de-identified medical records with daily integration of new patient data, standardized using terminologies including ICD-10 and LOINC (Logical Observation Identifiers Names and Codes). Clinical data extraction employs natural language processing with comprehensive quality assurance protocols ensuring database reliability. Patient privacy protection is maintained through aggregate data reporting and statistical summaries.

### Study outcomes

The outcomes included the need for rescue surgical intervention and mortality at 6 months (**Supplemental Table ****1**). Rescue surgery was operationally defined as any additional surgical drainage of a subdural hematoma performed after the index procedure within the 6-month follow-up window, identified using the same ICD-10-PCS procedure codes used to capture the index surgical evacuation.

### Statistical analysis

All statistical analyses were conducted within the TriNetX platform environment. Univariate comparisons utilized chi-square and Student’s t-tests as appropriate. Propensity score matching employed 1:1 nearest neighbor methodology incorporating demographic variables (age, sex, race, ethnicity), comorbidities (transient ischemic attack, cerebral infarction, hypertension, diabetes, alcohol use, tobacco use, obesity, chronic kidney disease, atrial fibrillation, ischemic heart disease, pulmonary embolism, deep venous thrombosis), and concurrent anticoagulation. Matching utilized greedy nearest neighbor algorithms with caliper width set at 0.1 pooled standard deviation. Covariate balance assessment employed standardized mean differences, with values exceeding 0.1 indicating inadequate balance. Statistical significance was established at two-sided α < 0.05. Effect estimates were expressed as odds ratios (OR) with 95% confidence intervals (95% CI). The TriNetX platform employs logistic regression for propensity score generation with randomized row ordering to minimize nearest neighbor matching bias.

## Results

Our analysis identified 179 patients who received combined surgery and MMAE and were initiated on antiplatelet therapy, compared to 2,392 patients who received surgery alone and were initiated on antiplatelet therapy. Additionally, 806 patients undergoing surgery with adjunct MMAE did not receive antiplatelet therapy during this period.

### Surgery with adjunct MMAE: Antiplatelet initiation versus non-initiation

After propensity score matching, 163 patients were included in each treatment arm (antiplatelet initiation versus non-initiation within the surgery with adjunct MMAE cohort). Pre-matching analysis revealed that patients receiving antiplatelet therapy demonstrated higher prevalence of cerebral infarction (24.6% vs. 11.7%, *P* < 0.001), essential hypertension (83.8% vs. 75.6%, *P* = 0.018), diabetes mellitus (43.6% vs. 34.6%, *P* = 0.024), chronic kidney disease (30.2% vs. 20.0%, *P* = 0.003), atrial fibrillation (30.7% vs. 23.6%, *P* = 0.045), and ischemic heart disease (55.3% vs. 29.2%, *P* < 0.001) (Table 1). Post-matching analysis demonstrated adequate covariate balance across treatment groups.

**Table 1:** Surgery with Adjunct MMAE - Demographics and Baseline Characteristics for Surgery with Adjunct MMAE Cohort, Stratified by Antiplatelet Initiation. MMAE, middle meningeal artery embolization; PSM, propensity score matching; Std Diff, standardized difference; TIA, transient ischemic attack; OR, odds ratio; CI, confidence interval

Characteristic	Before Matching				After Matching			
	**Antiplatelets** (*n* = 179)	**No Antiplatelets** (*n* = 806)	*P*-Value	Std Diff	**Antiplatelets** (*n* = 163)	**No Antiplatelets** (*n* = 163)	*P*-Value	Std Diff
**Demographics**								
Age at Index, years	72.5 ± 13.0	71.4 ± 12.5	0.317	0.082	72.9 ± 12.6	73.6 ± 11.8	0.592	0.059
Female	38 (21.2%)	210 (26.1%)	0.178	0.114	36 (22.1%)	38 (23.3%)	0.791	0.029
Male	139 (77.7%)	579 (71.8%)	0.113	0.134	126 (77.3%)	124 (76.1%)	0.793	0.029
**Race/Ethnicity**								
Black or African American	17 (9.5%)	96 (11.9%)	0.359	0.078	15 (9.2%)	22 (13.5%)	0.222	0.136
White	127 (70.9%)	498 (61.8%)	0.021	0.195	115 (70.6%)	107 (65.6%)	0.342	0.105
American Indian or Alaska Native	10 (5.6%)	10 (1.2%)	< 0.001	0.241	10 (6.1%)	10 (6.1%)	1.000	< 0.001
Asian	18 (10.1%)	104 (12.9%)	0.296	0.089	17 (10.4%)	16 (9.8%)	0.854	0.020
Hispanic or Latino	19 (10.6%)	53 (6.6%)	0.060	0.144	17 (10.4%)	12 (7.4%)	0.331	0.108
**Comorbidities**								
Cerebral Infarction	44 (24.6%)	94 (11.7%)	< 0.001	0.340	37 (22.7%)	41 (25.2%)	0.604	0.058
Essential Hypertension	150 (83.8%)	609 (75.6%)	0.018	0.206	134 (82.2%)	133 (81.6%)	0.886	0.016
Diabetes Mellitus	78 (43.6%)	279 (34.6%)	0.024	0.184	69 (42.3%)	63 (38.7%)	0.498	0.075
History of Nicotine Dependence	65 (36.3%)	240 (29.8%)	0.087	0.139	57 (35.0%)	55 (33.7%)	0.816	0.026
Overweight and Obesity	48 (26.8%)	190 (23.6%)	0.359	0.075	42 (25.8%)	39 (23.9%)	0.701	0.043
Chronic Kidney Disease	54 (30.2%)	161 (20.0%)	0.003	0.237	48 (29.4%)	36 (22.1%)	0.129	0.169
Atrial Fibrillation and Flutter	55 (30.7%)	190 (23.6%)	0.045	0.161	48 (29.4%)	50 (30.7%)	0.809	0.027
Asthma	13 (7.3%)	58 (7.2%)	0.975	0.003	10 (6.1%)	14 (8.6%)	0.396	0.094
Alcohol Dependence	10 (5.6%)	41 (5.1%)	0.785	0.022	10 (6.1%)	10 (6.1%)	1.000	< 0.001
Current Nicotine Dependence	22 (12.3%)	77 (9.6%)	0.271	0.088	21 (12.9%)	11 (6.7%)	0.063	0.207
Ischemic Heart Disease	99 (55.3%)	235 (29.2%)	< 0.001	0.549	85 (52.1%)	89 (54.6%)	0.657	0.049
Pulmonary Embolism	10 (5.6%)	43 (5.3%)	0.893	0.011	10 (6.1%)	10 (6.1%)	1.000	< 0.001
History of TIA or Cerebral Infarction	39 (21.8%)	113 (14.0%)	0.009	0.204	33 (20.2%)	39 (23.9%)	0.423	0.089
History of Venous Thromboembolism	19 (10.6%)	64 (7.9%)	0.244	0.092	14 (8.6%)	23 (14.1%)	0.116	0.175
**Anticoagulant Medications**								
Enoxaparin	73 (40.8%)	194 (24.1%)	< 0.001	0.363	64 (39.3%)	62 (38.0%)	0.820	0.025
Warfarin	15 (8.4%)	64 (7.9%)	0.845	0.016	12 (7.4%)	19 (11.7%)	0.186	0.147
Apixaban	16 (8.9%)	78 (9.7%)	0.761	0.025	15 (9.2%)	15 (9.2%)	1.000	< 0.001
Rivaroxaban	10 (5.6%)	26 (3.2%)	0.128	0.115	10 (6.1%)	10 (6.1%)	1.000	< 0.001
Argatroban	0 (0%)	10 (1.2%)	0.134	0.159	0 (0%)	0 (0%)	--	--
Bivalirudin	10 (5.6%)	10 (1.2%)	< 0.001	0.241	10 (6.1%)	10 (6.1%)	1.000	< 0.001
Eptifibatide	10 (5.6%)	10 (1.2%)	< 0.001	0.241	0 (0%)	10 (6.1%)	0.001	0.362

No statistically significant differences between antiplatelet-treated and non-treated patients within the combined surgery and MMAE cohort (Table 2). Rescue surgical intervention rates were comparable between groups (7.4% vs. 10.4%; OR 0.68, 95% CI 0.32–1.48, *P* = 0.33), as were the mortality rates (11.7% vs. 8.0%; OR 1.52, 95% CI 0.73–3.20, *P* = 0.26).

**Table 2:** 6-Month Clinical Outcomes, Surgery with Adjunct MMAE Cohort, Stratified by Antiplatelet Initiation (after matching, *n* = 163 per group). OR, odds ratio; CI, confidence interval. Calculated from after-matching cohorts

Outcome	Antiplatelets (*n* = 163)	No Antiplatelets (*n* = 163)	*P*-value	OR (95% CI)
Rescue Surgery	12 (7.4%)	17 (10.4%)	0.33	0.68 (0.32–1.48)
6-Month Mortality	19 (11.7%)	13 (8.0%)	0.26	1.52 (0.73–3.20)

### Antiplatelet-treated patients: Surgery with adjunct MMAE versus surgery alone

Among antiplatelet-treated patients, propensity score matching yielded 176 patients in each treatment arm (surgery with adjunct MMAE versus surgery alone), with well-balanced baseline characteristics (Table 3). Comparative analysis revealed similar rescue surgery rates between cohorts (7.4% vs. 9.1%; OR 0.80, 95% CI 0.37–1.71, *P* = 0.56). Notably, the combined surgery and MMAE group demonstrated significantly reduced mortality compared to the surgery-only group (10.8% vs. 21.0%; OR 0.46, 95% CI 0.25–0.83, *P* = 0.009) (Table 4).

**Table 3:** Antiplatelet Initiation - Demographics and Baseline Characteristics for Antiplatelet-Treated Patients, Surgery with Adjunct MMAE Versus Surgery Alone. MMAE, middle meningeal artery embolization; PSM, propensity score matching; Std Diff, standardized difference; TIA, transient ischemic attack; OR, odds ratio; CI, confidence interval

Characteristic	Before Matching				After Matching			
	**Surgery + MMAE** (*N* = 179)	**Surgery Only** (*N* = 2,*392)*	*P*-Value	Std Diff	**Surgery + MMAE** (*N* = 176)	**Surgery Only** (*N* = 176)	*P*-Value	Std Diff
**Demographics**								
Age at Index, years	72.5 ± 13.0	69.8 ± 14.1	0.016	0.194	72.2 ± 12.9	73.9 ± 12.0	0.203	0.136
Female	38 (21.2%)	702 (29.4%)	0.020	0.188	37 (21.0%)	35 (19.9%)	0.792	0.028
Male	139 (77.7%)	1,627 (68.0%)	0.007	0.217	137 (77.8%)	139 (79.0%)	0.796	0.028
**Race/Ethnicity**								
Black or African American	17 (9.5%)	280 (11.7%)	0.372	0.072	17 (9.7%)	16 (9.1%)	0.855	0.019
White	127 (70.9%)	1,682 (70.3%)	0.865	0.013	126 (71.6%)	130 (73.9%)	0.632	0.051
American Indian or Alaska Native	10 (5.6%)	30 (1.3%)	< 0.001	0.240	10 (5.7%)	0 (0%)	0.001	0.347
Asian	18 (10.1%)	123 (5.1%)	0.005	0.186	16 (9.1%)	18 (10.2%)	0.718	0.038
Hispanic or Latino	19 (10.6%)	167 (7.0%)	0.071	0.128	142 (80.7%)	144 (81.8%)	0.785	0.029
**Comorbidities**								
Cerebral Infarction	44 (24.6%)	465 (19.4%)	0.096	0.124	41 (23.3%)	38 (21.6%)	0.702	0.041
Essential Hypertension	150 (83.8%)	1,911 (79.9%)	0.210	0.101	148 (84.1%)	142 (80.7%)	0.401	0.090
Diabetes Mellitus	78 (43.6%)	1,006 (42.1%)	0.695	0.030	77 (43.8%)	77 (43.8%)	1.000	< 0.001
History of Nicotine Dependence	65 (36.3%)	707 (29.6%)	0.058	0.144	64 (36.4%)	64 (36.4%)	1.000	< 0.001
Overweight and Obesity	48 (26.8%)	498 (20.8%)	0.059	0.141	46 (26.1%)	46 (26.1%)	1.000	< 0.001
Chronic Kidney Disease	54 (30.2%)	587 (24.6%)	0.094	0.126	52 (29.5%)	52 (29.5%)	1.000	< 0.001
Atrial Fibrillation and Flutter	55 (30.7%)	704 (29.4%)	0.717	0.028	55 (31.3%)	60 (34.1%)	0.570	0.061
Asthma	13 (7.3%)	192 (8.0%)	0.715	0.029	12 (6.8%)	14 (8.0%)	0.684	0.043
Alcohol Dependence	10 (5.6%)	161 (6.7%)	0.553	0.048	10 (5.7%)	10 (5.7%)	1.000	< 0.001
Current Nicotine Dependence	22 (12.3%)	290 (12.1%)	0.949	0.005	22 (12.5%)	17 (9.7%)	0.396	0.091
Ischemic Heart Disease	99 (55.3%)	1,291 (54.0%)	0.734	0.026	97 (55.1%)	107 (60.8%)	0.280	0.115
Pulmonary Embolism	10 (5.6%)	106 (4.4%)	0.473	0.053	10 (5.7%)	10 (5.7%)	1.000	< 0.001
History of TIA or Cerebral Infarction	39 (21.8%)	502 (21.0%)	0.802	0.019	39 (22.2%)	44 (25.0%)	0.530	0.067
History of Venous Thromboembolism	19 (10.6%)	209 (8.7%)	0.395	0.063	18 (10.2%)	21 (11.9%)	0.610	0.054
**Anticoagulant Medications**								
Enoxaparin	73 (40.8%)	768 (32.1%)	0.017	0.181	70 (39.8%)	65 (36.9%)	0.584	0.058
Warfarin	15 (8.4%)	298 (12.5%)	0.107	0.134	15 (8.5%)	11 (6.3%)	0.415	0.087
Apixaban	16 (8.9%)	174 (7.3%)	0.413	0.061	15 (8.5%)	22 (12.5%)	0.224	0.130
Rivaroxaban	10 (5.6%)	86 (3.6%)	0.176	0.095	10 (5.7%)	10 (5.7%)	1.000	< 0.001
Argatroban	0 (0%)	10 (0.4%)	0.386	0.092	0 (0%)	0 (0%)	--	--
Bivalirudin	10 (5.6%)	66 (2.8%)	0.031	0.142	10 (5.7%)	10 (5.7%)	1.000	< 0.001
Eptifibatide	10 (5.6%)	14 (0.6%)	< 0.001	0.292	10 (5.7%)	10 (5.7%)	1.000	< 0.001

**Table 4:** 6-Month Clinical Outcomes, Antiplatelet-Treated Patients, Surgery with Adjunct MMAE Versus Surgery Alone (after matching, *n* = 176 per group). OR, odds ratio; CI, confidence interval. Calculated from after-matching cohorts

Outcome	Surgery + MMAE (*n* = 176)	Surgery Only (*n* = 176)	*P*-value	OR (95% CI)
Rescue Surgery	13 (7.4%)	16 (9.1%)	0.56	0.80 (0.37–1.71)
6-Month Mortality	19 (10.8%)	37 (21.0%)	0.009	0.46 (0.25–0.83)

## Discussion

This multi-institutional analysis provides new insight into the management of antiplatelet therapy in cSDH patients, particularly in the context of adjunctive MMAE. We found that reinitiating antiplatelet agents within one month of cSDH evacuation did not lead to higher rates of hematoma recurrence or rescue surgical intervention in patients treated with combined surgery and MMAE. In fact, the odds of requiring a reoperation were statistically similar whether or not antiplatelet therapy was resumed early, and mortality did not differ significantly between these groups. Furthermore, among patients who resumed antiplatelet therapy postoperatively, those who had received adjunct MMAE experienced comparable reoperation rates (7.4% vs. 9.1%, respectively) to those treated with surgery alone, but notably lower 6-month mortality rates (10.8% vs. 21%, respectively). This observation suggests a potential survival benefit associated with MMAE in high-risk patients on antiplatelet therapy, underscoring that adding MMAE may improve outcomes without incurring additional risk of rebleeding. This mortality benefit pattern has been observed consistently across different cSDH populations, including recent real-world data showing similar survival advantages with MMAE in nonsurgical patients managed conservatively, suggesting that MMAE’s therapeutic effects extend beyond hemorrhage prevention [23]. Overall, our findings indicate that early postoperative antiplatelet resumption is feasible and safe in cSDH patients, particularly when MMAE is utilized, challenging the common practice of prolonged antithrombotic cessation.

These results align with the emerging literature on the fact that the hemorrhagic risk following the resumption of antithrombotic management in cSDH may be overestimated. Martinez-Gutierrez et al. reported favorable outcomes in cSDH patients undergoing MMAE while on antithrombotic therapy, supporting its safety in anticoagulated and antiplatelet-treated individuals populations [17]. The recent randomized SECA trial found no significant difference in 6-month cSDH recurrence between patients who continued aspirin through the perioperative period and those who discontinued it (13.9% vs. 9.5%, *P* = 0.32) [24]. This corresponds with prior meta-analyses of non-embolization cohorts showing that resumption of antithrombotic agents does not significantly elevate recurrence rates [16, 25]. Wang et al. reported no significant difference in recurrence rates between patients who resumed antithrombotics and those who did not (15.0% vs. 19.4%, *P* = 0.55) [26], while a comprehensive meta-analysis by Phan et al. showed similar findings with pooled recurrence rates of 14.8% versus 18.7% (*P* = 0.18) for resumed versus non-resumed therapy, respectively [25]. The German multicenter study by Hamou et al. provided critical evidence demonstrating that discontinuation of antithrombotic therapy led to thrombotic complications in 9.1% of patients, with an odds ratio of 8.28 (95% CI 1.8-38.12, *P* = 0.007) for previously anticoagulated patients [27]. Furthermore, a 2025 meta-analysis of antithrombotic resumption after MMAE involving 233 patients found no significant difference in recurrence rates (OR 1.64, 95% CI 0.45-6.00, *P* = 0.45) between patients who resumed therapy and those who did not [16]. Consistent with these findings, our study demonstrates that early antiplatelet reinitiation after cSDH evacuation did not worsen outcomes in the surgery with the adjunctive MMAE group. In fact, by preventing the cSDH from reaccumulating, MMAE might neutralize the theoretical pro-hemorrhagic effect of antiplatelets.

Adjunctive MMAE has been proven in randomized trials to dramatically reduce cSDH recurrence risk. For instance, the EMBOLISE trial reported a threefold reduction in rescue surgery with MMAE (approximately 4% vs. 11% within 90 days), by occluding the fragile neovasculature thought to drive recurrent bleeding [11]. Our real-world data suggest that harnessing this benefit of MMAE allows patients to restart needed antiplatelet therapy early without incurring additional hematoma growth. Supporting this notion, a recent systematic review of cSDH patients treated with MMAE (with or without surgery) likewise found no increase in recurrence or slower hematoma resolution among those who resumed antithrombotics compared to those who did not [16]. Chaliparambil et al. even reported no significant difference in 3-month radiographic hematoma resolution with post-embolization antithrombotic use, reinforcing that restarting such therapy appears safe in the setting of MMAE [28]. Additionally, a systematic review by Alkhiri et al., encompassing 16 studies and 4,606 patients, found that 69.0% of patients safely resumed antithrombotic therapy with bleeding complication rates of only 14.1% (95% CI 9.7–20.2%), comparable to non-resumed therapy groups [9]. Taken together, the growing body of evidence, including our current study, suggests that routine prolonged discontinuation of antiplatelet agents after cSDH may be unnecessary, especially when modern adjuncts, such as MMAE, are employed to mitigate the risk of rebleeding.

Equally important are the implications for preventing ischemic complications in this often elderly, co-morbid patient population. Patients with cSDH frequently have underlying cardiovascular or cerebrovascular disease (e.g., atrial fibrillation, coronary artery disease, prior stroke), meaning that interrupting antithrombotic therapy puts them at real danger of thromboembolic events [27, 29]. In fact, one study showed that failure to resume antiplatelet therapy after cSDH surgery increased the risk of stroke or myocardial infarction by 7.5-fold. Our findings that early resumption of antiplatelets can be accomplished without exacerbating hemorrhagic outcomes are therefore clinically significant, namely a more balanced approach that minimizes time off critical antithrombotic medications. By safely mitigating the competing risks, adjunctive MMAE may provide a means to protect against both hematoma recurrence and thromboembolic events. Although our analysis did not directly measure ischemic outcomes, the ability to reinstitute antiplatelet therapy promptly should in theory, translate to fewer cardiovascular complications. Indeed, preliminary evidence suggests that both antiplatelet and anticoagulant agents can be restarted early after cSDH treatment with MMAE without impacting hematoma recovery [16, 19]. This paradigm shift is supported by recent trial data indicating that adding MMAE to standard cSDH treatment does not increase adverse events such as stroke, while significantly reducing the need for additional interventions [13]. In essence, the adjunct of MMAE appears to provide a safety net that addresses neurosurgeons’ bleeding concerns, thereby permitting timely prophylaxis against strokes and other thromboses in high-risk patients.

The observed survival difference in our antiplatelet-treated MMAE cohort is hypothesis-generating and warrants careful interpretation. At 6 months post-treatment, mortality was roughly halved in patients who underwent surgery with adjunct MMAE compared to those who had surgery alone while on antiplatelet therapy. This difference (10.8% vs. 21.0%) was statistically significant and may reflect improved overall outcomes when MMAE is incorporated. One plausible explanation is that by reducing occult rebleeding and chronic hematoma persistence, MMAE helps patients avoid the potential complications (neurological decline, rescue surgeries, prolonged hospitalization) that can drive mortality in cSDH. Even though our matched comparison did not show a lower reoperation rate within 6 months for the MMAE group (perhaps due to limited sample size or the offsetting influence of anticoagulant/antiplatelet use on both cohorts), the embolization might still confer subtler benefits, namely better hematoma resolution, less subdural membrane proliferation, or improved neurological recovery, that enhance patient resilience. It is also possible that the mortality disparity is partly attributable to unmeasured confounders. Patients selected for MMAE, even after matching for comorbidities, may have had differences in hematoma characteristics or care environments that influenced outcomes [30]. Nevertheless, it is reassuring that, in randomized trials, the addition of MMAE did not result in any increase in serious complications or early mortality [11, 13, 14]. In our study, the fact that adjunctive MMAE was associated with lower mortality (and certainly no increase) suggests that its net effect on patient outcomes appears favorable, although this association is observational and residual confounding cannot be excluded.

### Limitations

Despite its strengths, this study has several limitations. It was a retrospective observational analysis of electronic health records and is therefore subject to coding inaccuracies, selection bias, and residual confounding. We used ICD-10 and procedure codes to identify cSDH, surgical evacuation, and MMAE; however, coding practices may vary across institutions and could affect case ascertainment and outcome classification. Important clinical variables were unavailable, including hematoma size, chronicity, laterality, midline shift, radiographic subtype, surgical technique, intraoperative irrigation, drain use and duration, timing of antiplatelet resumption, and the clinical indication for restarting antiplatelets. These factors may influence recurrence and outcomes but could not be captured in the TriNetX federated dataset, which relies on structured coded data and does not include granular operative details or imaging-derived measurements. Although we matched for demographics and comorbidities and excluded patients receiving full anticoagulation, unmeasured confounding remains possible. The decision to restart antiplatelet therapy was not randomized and likely reflected individualized clinician assessment of thrombotic and hemorrhagic risk, introducing treatment selection bias. In addition, while we evaluated antiplatelet reinitiation within one month after surgery, the dataset did not allow identification of a more precise timing window or an optimal threshold for safe resumption. Single-center data were also explored, but the small number of patients with post-treatment antiplatelet reinitiation precluded meaningful analysis, highlighting the need for larger multicenter or imaging-linked registries.

Despite these limitations, this study provides rare large-scale evidence on the safety of antiplatelet reinitiation in patients with cSDH treated with surgical evacuation and adjunct MMAE. The findings suggest that, in selected high-risk patients requiring antiplatelet therapy for vascular protection, early resumption after hematoma evacuation and MMAE may be feasible without worsening hematoma-related outcomes. This strategy may help balance the recurrence-reducing effect of MMAE with the need to minimize prolonged interruption of antithrombotic therapy.

## Conclusion

Reinitiating antiplatelet therapy within one month after surgical evacuation of a cSDH did not increase rebleeding or reoperation rates in our matched cohort, especially when combined with MMAE. Adjunctive MMAE was associated with comparable rescue surgery rates and lower 6-month mortality in antiplatelet-treated patients compared with surgery alone, although this association is observational and hypothesis-generating, and residual confounding cannot be excluded. These findings suggest that the addition of MMAE may provide a margin of safety that supports the timely resumption of antiplatelet therapy in patients who require it, without an apparent increase in hemorrhagic complications. Given the high thromboembolic risk of withholding antithrombotics in this population, our study supports the practice of early postoperative antiplatelet reinstatement when clinically indicated, provided that diligent hematoma management (potentially including MMAE) is in place. Further Prospective and randomized studies are necessary to verify these results and determine the best timing and protocols.

## Supplementary Information

Below is the link to the electronic supplementary material.

Supplementary Material 1

## Data Availability

No datasets were generated or analysed during the current study.

## Abbreviations

cSDH: chronic subdural hematoma
MMAE: middle meningeal artery embolization
PSM: propensity score matching
SDH: subdural hematoma
TIA: transient ischemic attack
OR: odds ratio
CI: confidence interval
ICD: 10-International Classification of Diseases, 10th revision
PCS: Procedure Coding System
RXNORM: RxNorm terminology
UMLS: Unified Medical Language System

## Declarations

### Guarantor

Dhairya A Lakhani, MD.
