# Supplementary material for: Reinitiating antiplatelet therapy in chronic subdural hematoma: Does adjunctive middle meningeal artery embolization improve outcomes?
Source: Neurosurg Rev. 2026 May 9;49(1):398. doi: 10.1007/s10143-026-04318-6 (PMC13156191; doi:10.1007/s10143-026-04318-6)
Supplement: Supplementary file 1 — Supplementary Material 1 [file 10143_2026_4318_MOESM1_ESM.docx]

**Supplemental Table 1:** ICD-10 and RXNORM Codes Used for Patient Identification and Outcome Definitions

| **Code** | **Description** |
| --- | --- |
| **cSDH diagnosis** | |
| UMLS:ICD10CM:I62.02 | Nontraumatic subacute subdural hemorrhage |
| UMLS:ICD10CM:I62.00 | Nontraumatic subdural hemorrhage, unspecified |
| UMLS:ICD10CM:I62.03 | Nontraumatic chronic subdural hemorrhage |
| **Surgery** | |
| UMLS:ICD10PCS:00C43ZZ | Extirpation of Matter from Intracranial Subdural Space, Percutaneous Approach |
| UMLS:ICD10PCS:009400Z | Drainage of Intracranial Subdural Space with Drainage Device, Open Approach |
| UMLS:ICD10PCS:00C44ZZ | Extirpation of Matter from Intracranial Subdural Space, Percutaneous Endoscopic Approach |
| UMLS:ICD10PCS:009430Z | Drainage of Intracranial Subdural Space with Drainage Device, Percutaneous Approach |
| UMLS:ICD10PCS:00943ZZ | Drainage of Intracranial Subdural Space, Percutaneous Approach |
| UMLS:ICD10PCS:009440Z | Drainage of Intracranial Subdural Space with Drainage Device, Percutaneous Endoscopic Approach |
| UMLS:ICD10PCS:00C40ZZ | Extirpation of Matter from Intracranial Subdural Space, Open Approach |
| UMLS:ICD10PCS:00944ZZ | Drainage of Intracranial Subdural Space, Percutaneous Endoscopic Approach |
| UMLS:ICD10PCS:00940ZZ | Drainage of Intracranial Subdural Space, Open Approach |
| **MMAE** | |
| UMLS:ICD10PCS:03LG3DZ | Occlusion of Intracranial Artery with Intraluminal Device, Percutaneous Approach |
| **Antiplatelets** | |
| NLM:RXNORM:1191 | aspirin |
| NLM:RXNORM:32968 | clopidogrel |
| NLM:RXNORM:1656052 | cangrelor |
| NLM:RXNORM:3521 | dipyridamole |
| NLM:RXNORM:1116632 | ticagrelor |
| NLM:RXNORM:1537034 | vorapaxar |
| NLM:RXNORM:613391 | prasugrel |
| NLM:RXNORM:10594 | ticlopidine |
| **Inpatient readmission** | |
| UMLS:HL7V3.0:VisitType:IMP | Visit: Inpatient Encounter |
| **Repeat acute or subacute SDH** | |
| UMLS:ICD10CM:I62.01 | Nontraumatic acute subdural hemorrhage |
| UMLS:ICD10CM:I62.02 | Nontraumatic subacute subdural hemorrhage |

cSDH, chronic subdural hematoma; MMAE, middle meningeal artery embolization; SDH, subdural hematoma
